# Assessment of Stress and Well-Being of Japanese Employees Using Wearable Devices for Sleep Monitoring Combined With Ecological Momentary Assessment: Pilot Observational Study

**DOI:** 10.2196/49396

**Published:** 2024-05-02

**Authors:** Shotaro Kinoshita, Sayaka Hanashiro, Shiori Tsutsumi, Kiko Shiga, Momoko Kitazawa, Yasuyo Wada, Jun Inaishi, Kazuhiro Kashiwagi, Toshikazu Fukami, Yasumasa Mashimo, Kazumichi Minato, Taishiro Kishimoto

**Affiliations:** 1 Hills Joint Research Laboratory for Future Preventive Medicine and Wellness Keio University School of Medicine Tokyo Japan; 2 Graduate School of Interdisciplinary Information Studies The University of Tokyo Tokyo Japan; 3 Department of Neuropsychiatry Keio University School of Medicine Tokyo Japan; 4 Graduate School of Health Management Keio University Kanagawa Japan; 5 Department of Clinical Psychology, Faculty of Human Relations Shigakukan University Kagoshima Japan; 6 Center for Preventice Medicine Keio University Hospital Tokyo Japan; 7 Department of Health Promotion National Institute of Public Health Saitama Japan; 8 Division of Endocrinology, Metabolism and Nephrology, Department of Internal Medicine Keio University School of Medicine Tokyo Japan; 9 TechDoctor, Inc. Tokyo Japan; 10 Department of Psychiatry The Zucker Hillside Hospital, Northwell Health New York, NY United States; 11 Department of Psychiatry Donald and Barbara Zucker School of Medicine at Hofstra/Northwell Hempstead, NY United States; 12 Department of Molecular Medicine Donald and Barbara Zucker School of Medicine at Hofstra/Northwell Hempstead, NY United States

**Keywords:** wearable device, sleep feedback, well-being, stress, ecological momentary assessment, feasibility study

## Abstract

**Background:**

Poor sleep quality can elevate stress levels and diminish overall well-being. Japanese individuals often experience sleep deprivation, and workers have high levels of stress. Nevertheless, research examining the connection between objective sleep assessments and stress levels, as well as overall well-being, among Japanese workers is lacking.

**Objective:**

This study aims to investigate the correlation between physiological data, including sleep duration and heart rate variability (HRV), objectively measured through wearable devices, and 3 states (sleepiness, mood, and energy) assessed through ecological momentary assessment (EMA) and use of rating scales for stress and well-being.

**Methods:**

A total of 40 office workers (female, 20/40, 50%; mean age 40.4 years, SD 11.8 years) participated in the study. Participants were asked to wear a wearable wristband device for 8 consecutive weeks. EMA regarding sleepiness, mood, and energy levels was conducted via email messages sent by participants 4 times daily, with each session spaced 3 hours apart. This assessment occurred on 8 designated days within the 8-week timeframe. Participants’ stress levels and perception of well-being were assessed using respective self-rating questionnaires. Subsequently, participants were categorized into quartiles based on their stress and well-being scores, and the sleep patterns and HRV indices recorded by the Fitbit Inspire 2 were compared among these groups. The Mann-Whitney *U* test was used to assess differences between the quartiles, with adjustments made for multiple comparisons using the Bonferroni correction. Furthermore, EMA results and the sleep and HRV indices were subjected to multilevel analysis for a comprehensive evaluation.

**Results:**

The EMA achieved a total response rate of 87.3%, while the Fitbit Inspire 2 wear rate reached 88.0%. When participants were grouped based on quartiles of well-being and stress-related scores, significant differences emerged. Specifically, individuals in the lowest stress quartile or highest subjective satisfaction quartile retired to bed earlier (*P*<.001 and *P*=.01, respectively), whereas those in the highest stress quartile exhibited greater variation in the midpoint of sleep (*P*<.001). A multilevel analysis unveiled notable relationships: intraindividual variability analysis indicated that higher energy levels were associated with lower deviation of heart rate during sleep on the preceding day (β=–.12, *P*<.001), and decreased sleepiness was observed on days following longer sleep durations (β=–.10, *P*<.001). Furthermore, interindividual variability analysis revealed that individuals with earlier midpoints of sleep tended to exhibit higher energy levels (β=–.26, *P*=.04).

**Conclusions:**

Increased sleep variabilities, characterized by unstable bedtime or midpoint of sleep, were correlated with elevated stress levels and diminished well-being. Conversely, improved sleep indices (eg, lower heart rate during sleep and earlier average bedtime) were associated with heightened daytime energy levels. Further research with a larger sample size using these methodologies, particularly focusing on specific phenomena such as social jet lag, has the potential to yield valuable insights.

**Trial Registration:**

UMIN-CTR UMIN000046858; https://center6.umin.ac.jp/cgi-open-bin/ctr/ctr_view.cgi?recptno=R000053392

## Introduction

Sleep is one of the most important health behaviors for human beings, and a lack of it affects both physical and mental health [1,2]. Furthermore, subjective stress and well-being are closely related to sleep quality [3-5].

Subjective methods for evaluating sleep quality are the Consensus Sleep Diary [6] and Pittsburgh Sleep Quality Index [7]; however, their results sometimes differ from those of objective assessments [7,8]. Therefore, objective sleep evaluation methods have been developed, and research using these methods is ongoing.

Polysomnography is the gold standard for objective sleep assessment methods [9], but it is expensive and physically demanding. Hence, wearable devices have emerged as recent alternatives for sleep assessment, offering less burdensome options compared with polysomnography [10]. For example, commercially available devices such as Fitbit (Fitbit Inc.) can measure motion, heart rate variability (HRV), and respiratory rate, and based on these measurements, sleep quality can be measured with reasonable accuracy [11]. Furthermore, HRV, a quantitative biomarker of autonomic activity [12], has been reported to be useful for the quantitative assessment of stress [13] and is highly associated with subjective well-being [14,15].

In addition, self-administered questionnaires such as the Epworth Sleepiness Scale have been used [16]; however, they do not capture diurnal variations in sleepiness. One method to capture symptom variability is ecological momentary assessment (EMA), which is a prospective, repeated sampling of symptoms in real time in the participants’ natural environment, with minimal burden on respondents [17]. EMA has been widely used to accurately assess respondents’ mood and stress levels during the day [18]. Moreover, several studies have examined sleep conditions and their relationship to daytime sleepiness using EMA [19-23]. In recent years, studies have combined objective sleep assessments, such as those obtained by wearables, with EMA-based assessments [24-26].

It has long been reported that, on average, Japanese people go to bed later and sleep for shorter durations than people in other countries [27,28]. A survey of 13 countries in 2020 and 2021 also reported that Japan had the lowest subjective satisfaction with sleep for 2 consecutive years [29]. In Japan, the number of individuals with work-related mental health problems continues to increase annually [30]; furthermore, in a 2023 survey on workers’ health and well-being, Japan ranked last among 30 countries [31]. However, only a few studies have evaluated the relationship between sleep and stress and well-being among Japanese workers using a combination of objective sleep assessments and EMA.

This study investigated the relationship between sleep and stress and well-being among Japanese workers. We evaluated the relationship between sleep quality/daytime sleepiness and stress levels/perception of well-being (hereinafter, stress/well-being) by combining the assessment of sleep quality using wearable devices with real-time monitoring of daytime sleepiness and mental status using EMA. In particular, we examined the relationships between objectively measured sleep indices and HRV using Fitbit Inspire 2 (Fitbit Inc.) and the participants’ subjective conditions or feelings to verify the following hypotheses: (1) people with short total sleep time and late bedtime have high stress levels and low perceived well-being; (2) people with high sleep quality have high energy and good mood during the day.

## Methods

### Study Design

The study was designed to explore the correlations between physiological and behavioral data (eg, sleep, daytime activity, HRV, and blood glucose) objectively measured by wearable devices, and the participants’ perception of well-being, stress levels, daily mood, energy, and sleepiness assessed through questionnaires and EMA. The participants were asked to wear 2 wearable devices, namely, the Fitbit Inspire 2 and the FreeStyle Libre (Abbott Diabetes Care) for acquiring the physiological/behavioral data and 24-hour blood glucose data, respectively. In this study, our focus was on examining the relationships between sleep and HRV indices measured using the Fitbit Inspire 2, alongside the concurrent assessment of participants’ psychological status through EMA and stress/well-being questionnaires. Other aspects of the study will be reported separately.

The detailed study design consists of recruiting a total of 40 participants, comprising individuals with impaired glucose tolerance (not meeting the diagnostic criteria for diabetes mellitus) and healthy participants. Initially, participants completed a questionnaire aimed at evaluating their eating behaviors and stress/well-being. Following this, they were instructed to wear the Fitbit Inspire 2 continuously for 8 consecutive weeks. In addition, during the initial 2 weeks of the study, participants were instructed to wear the FreeStyle Libre continuously during the day. After the completion of this 2-week period, participants were asked to once again complete the questionnaire assessing their eating behaviors. Furthermore, they received guidance on eating behaviors from a dietitian. Following the initial 4 weeks of wearing the Fitbit Inspire 2 device, participants were requested to wear the FreeStyle Libre once more for a duration of 2 weeks. This period coincided with the final 2 weeks of the 8-week Fitbit Inspire 2 wearing period. Subsequently, upon concluding the 8-week duration of wearing both devices, participants were once again asked to complete the questionnaires assessing their eating behaviors, as well as another questionnaire aimed at evaluating their stress/well-being. Furthermore, EMA was conducted to evaluate sleepiness, mood, and energy levels. This assessment involved participants sending emails 4 times a day, with each email sent 3 hours apart. This process occurred on 8 designated days throughout the 8-week study period.

### Participants

The inclusion and exclusion criteria for participants are detailed in Textbox 1. Eligible participants were adults, defined as individuals at least 20 years old (the age of adulthood in Japan at the time of recruitment), who possessed a smartphone (either Android or iOS). The study aimed to investigate the correlation between sleep, HRV, 24-hour blood glucose levels, and various psychological factors including sleepiness, energy, mood, and stress/well-being. Therefore, inclusion criteria encompassed individuals expected to exhibit high blood glucose variability, alongside normal participants. Specifically, participants with abnormal glucose tolerance (hemoglobin A_1C_ [HbA_1c_] levels ranging from 5.8 to <6.5) who did not meet the criteria for diabetes mellitus were considered eligible. Psychiatric disorders were neither inclusion nor exclusion criteria for participant selection. Recruitment for the study occurred in February 2022, with participants recruited at a 1:1 male-to-female ratio.

**Inclusion and exclusion criteria.**

1. Inclusion criteria
  - Patients with hemoglobin A_1C_ (HbA_1c_) values of 5.8≤HbA_1c_<6.5 at the most recent physical examination undergone within the previous year, or healthy participants with no glucose intolerance.
  - Adults aged 20 years or older at the time of consent acquisition.
  - Smartphone users who owned and used a smartphone that was compatible with the app used in this study.
2. Exclusion criteria
  - Patients who had been diagnosed and treated for diabetes mellitus in the past.
  - Patients with comorbidities that could affect the data measured by the wearable wristband device, such as paralysis of the upper limbs.
  - Others who were deemed as being inappropriate participants for the study by the principal researcher or coresearchers.

### Data Collection

The data collected in this study are outlined comprehensively in Textbox 2. Further, the data collection schedule is presented in Table 1. Data collection occurred between February 2022 and April 2022. The Perceived Stress Scale (PSS) [32] was used for the assessment of subjective stress. For the assessment of subjective well-being, we used the Satisfaction With Life Scale (SWLS) [33], the Japanese version of the Scale of Positive and Negative Experience (SPANE-J) [34], and the Japanese version of the Flourishing Scale (FS-J) [34]. To evaluate sleepiness, the Japanese version of the Epworth Sleepiness Scale (JESS) [35] was used.

**Data collected in this study.**

1. Background information (results of company medical checkup)
  - Gender, age, and preexisting medical conditions
  - Results of the most recent medical checkup undergone within the previous year
2. Questionnaire
  - Brief-Type Self-Administered Diet History Questionnaire (BDHQ) [36]
  - Eating Behavior Questionnaire (EBQ) [37]
  - Perceived Stress Scale (PSS) [32]
  - Satisfaction With Life Scale (SWLS) [33]
  - The Japanese version of the Scale of Positive and Negative Experience (SPANE-J) [34]
  - The Japanese version of the Flourishing Scale (FS-J) [34]
  - The Japanese version of the Epworth Sleepiness Scale (JESS) [35]
3. Ecological momentary assessment
  - Sleepiness (Visual Analog Scale [VAS] format): scores range from 0=not sleepy at all to 100=very sleepy (maximum sleepiness imaginable).
  - Mood (VAS format): scores range from 0=negative mood (lowest mood imaginable) to 100=positive mood (best mood imaginable).
  - Energy (VAS format): scores range from 0=no energy at all to 100=full of energy (greatest state of energy imaginable).
4. Wearable Devices
  - Fitbit Inspire 2 data (eg, sleep, heart rate variability, and body movements)
  - FreeStyle Libre data (24-hour blood glucose)

**Table 1:** Schedule for data collection.^a^

Schedule	Baseline	2 weeks	Intermediate evaluation	Interval (4 weeks)	2 weeks	Final evaluation
Background information	✓					
Medical checkup	✓					
BDHQ^a^	✓		✓			✓
EBQ^b^	✓		✓			✓
PSS^c^	✓					✓
SWLS^d^	✓					✓
SPANE-J^e^	✓					✓
FS-J^f^	✓					✓
JESS^g^	✓					✓

^a^BDHQ: Brief-Type Self-Administered Diet History Questionnaire.

^b^EBQ: Eating Behavior Questionnaire.

^c^PSS: Perceived Stress Scale.

^d^SWLS: Satisfaction With Life Scale.

^e^SPANE-J: The Japanese version of the Scale of Positive and Negative Experience.

^f^FS-J: The Japanese version of the Flourishing Scale.

^g^JESS: The Japanese version of the Epworth Sleepiness Scale.

Fitbit Inspire 2 data were collected continuously throughout the 8-week study period. FreeStyle Libre data and EMA data were collected 2 weeks after baseline and 2 weeks before the final evaluation.

Data collection was facilitated through electronic patient-reported outcomes, a self-administered instrument completed by participants independently. Electronic patient-reported outcome response links were disseminated via email and accessed by participants through their smartphones. An identical method was used for EMA.

As previously noted, not all data from this study are presented within this paper. The results of the assessment of eating behaviors and blood glucose–related data, along with the corresponding analyses, were reported elsewhere [38,39].

### EMA

In the EMA, participants were prompted to respond to questions regarding 3 states: sleepiness (ranging from “not sleepy at all” to “very sleepy [maximum sleepiness imaginable]”), mood (ranging from “negative mood [lowest mood imaginable]” to “positive mood [maximum mood imaginable]”), and energy (ranging from “no energy at all” to “full of energy [greatest state of energy imaginable]”). These responses were provided using the Visual Analog Scale (VAS) format. The design of these VAS items was informed by previous studies that combined EMA with the VAS [40,41].

Throughout the study period, the questions were administered 2 times a week on weekdays, occurring on 2 nonconsecutive days (such as Tuesday and Thursday or Wednesday and Friday). These questions were delivered at defined times of the day, spaced 3 hours apart. Specifically, the questions were presented at 9 AM, noon, 3 PM, and 6 PM. Participants were then requested to respond to the questions during the following time intervals: 9 AM to noon, noon to 3 PM, 3 PM to 6 PM, and after 6 PM, respectively.

The EMA score data collected on the same day as the email was sent were used in the analysis. Fitbit Inspire 2 activity indicators, including heart rate, number of steps, and metabolic equivalents (METs), were aggregated within the 1-hour time window immediately preceding the EMA response time. Each EMA response is collected as time-stamped data, and the analysis is conducted retrospectively from the time stamp, specifically 1 hour before the EMA response time. Sleep indices were compiled from Fitbit Inspire 2 sleep records where the waking time fell within the time window from 6 PM on the day preceding the EMA response date to 9 AM on the EMA response date. In instances where multiple sleep records were present within this timeframe, the sleep periods were consolidated, and sleep indices were recalculated accordingly.

### Fitbit Inspire 2

Fitbit Inspire 2 is a commercially available device equipped with a triaxial acceleration sensor, which allows for the calculation of body movement, sleep patterns, and other related metrics. In addition, it features a pulse sensor that collects data for each heartbeat using the photoelectric pulse wave method. Participants were instructed to wear the Fitbit Inspire 2 device, distributed at the outset of the study, continuously for a designated period (8 weeks). Participants were permitted to temporarily remove the Fitbit Inspire 2 device for short durations, such as during bathing or when recharging the device. As the Fitbit Inspire 2 has a data storage capacity of approximately 7 days, participants were requested to periodically transmit the collected data to the cloud using their smartphones.

Fitbit Inspire 2 records were considered valid record days if at least 80% of the day was recorded. When matching against EMA data, only participants with a wear rate exceeding 80% in the hour preceding the most recent hour of EMA activity were included. For investigations into the relationship between EMA results and sleep, only participants with a wearing rate of 80% or greater between 6 PM on the day before the response date and 9 AM on the day of the response were included in the analysis.

### Statistical Analysis

All data collected during the study were incorporated into the analysis. We adopted an exploratory analysis approach, thoroughly examining the various relationships among the data obtained from the wearable devices, EMA, and assessments of stress/well-being.

The Fitbit Inspire 2 device provides data on time in bed (TIB), mid-wake time, and sleep duration. Using this information, we computed bedtime, midpoint of sleep, and sleep efficiency (calculated as 100 × sleep duration/TIB) for the analyses conducted in this study. However, we chose not to include sleep stage estimates provided by the device in our analyses because of concerns regarding their validation. Therefore, this parameter was excluded from our analysis. Based on the pulse data obtained from the Fitbit Inspire 2, we calculated the SD of the NN intervals (SDNN) to assess HRV. In analyzing the SDNN, we used a 15-minute time window. This decision was made considering that the pulse was measured once every 5 seconds. Using a shorter time window, such as 5 minutes, might lead to less reliable values because of the limited amount of data available. Although SDNN is typically calculated based on each pulse interval, the Fitbit Inspire 2 reports pulse rate data every 5 seconds. Therefore, in this study, the SDNN was calculated based on the pulse rate provided at 5-second intervals.

The participants were stratified into quartiles based on their scores on the stress/well-being questionnaires. We then compared the sleep and HRV indices measured by the Fitbit Inspire 2 between the groups with scores in the lowest and highest quartiles. To assess the differences between these 2 groups, we used the Mann–Whitney *U* test. Given the multiple comparisons conducted, we applied the Bonferroni correction to adjust for potential type I errors.

The study also investigated the relationships between the sleep and HRV indices measured by the Fitbit Inspire 2 and the daily mood, energy levels, and sleepiness assessed by EMA.

In the multilevel analysis, 3 types of multilevel models were constructed, with the EMA scores for energy, mood, and drowsiness serving as the dependent variables. The independent variables were selected through forward stepwise selection using the Akaike information criterion as the evaluation index. The sleep index, sleep heart rate, and sleep HRV index measured by the Fitbit Inspire 2 were considered potential candidates for the independent variables in the models. In addition to the main predictors, control variables such as age, sex, response time, and MET time were included in the analysis. However, to mitigate the influence of activity immediately preceding the response on the EMA score, the aggregate value of the MET time from 1 hour before the response to the time of the response was used. Sleep indices were separated into 2 components: the mean value across all participants (interindividual), such as average bedtime among participants, and within-participant deviations (intraindividual), such as bedtime deviations for each participant. These components were then used as explanatory variables in the analysis. The statistical analyses described above were performed using either Python (Python Foundation) or R (R Foundation) programming languages.

### Ethical Consideration

This study was carried out with the approval of the Keio University School of Medicine Ethics Committee (ID 20211103). Before enrollment, all participants provided written informed consent. All collected information was anonymized and used solely for the purpose of the study. Participants did not receive any monetary compensation; however, they were provided with feedback on their own data collected during the study.

## Results

### Participant Characteristics

A cohort of 40 eligible participants, comprising office workers from a real estate company, was enrolled in this study in February 2022. All participants successfully completed the entire study protocol. It is noteworthy that participants were required to work from their respective offices throughout the study duration, and none took extended vacations or engaged in remote work for prolonged periods during the study period. The demographic characteristics of the participants are summarized in Table 2. The mean age of the participants was 40.4 (SD 11.8) years, and the male-to-female ratio was 1:1. No individuals were excluded from participation due to meeting the exclusion criteria at the time of recruitment. Responses to the background information questionnaire revealed that none of the participants were currently undergoing treatment for psychiatric disorders. Furthermore, no serious adverse events, including instances of COVID-19 infection, were reported throughout the study duration. Histograms displaying the distribution of scores on the stress/well-being questionnaire within each quartile group are provided in Figures S1-S6 in Multimedia Appendix 1.

**Table 2:** Baseline characteristics of the participants in the study (N=40).

Characteristics	Value
Age (years), mean (SD)	40.4 (11.8)
Male, n (%)	20 (50)
Perceived Stress Scale (PSS), mean (SD)	19.2 (6.4)
Satisfaction With Life Scale (SWLS), mean (SD)	22.1 (6.1)
SPANE-J positive experience (SPANE-P), mean (SD)	20.7 (5.0)
SPANE-J negative experience (SPANE-N), mean (SD)	16.9 (4.5)
The Japanese version of the Flourishing Scale (FS-J), mean (SD)	39.8 (7.1)
The Japanese version of the Epworth Sleepiness Scale (JESS), mean (SD)	8.1 (4.0)

### EMA and Fitbit Inspire 2 Adherence

All participants successfully completed the program without dropping out. The overall response rate for EMA was 87.34% (1118/1280). Regarding the difference between EMA transmission time and response time, the median (IQR) was 11.8 (3.2-42.4) minutes. However, 1 participant was unaware of receiving emails until midway through the study period because the emails had been inadvertently sorted into the spam folder. The mean daily wear time for all Fitbit Inspire 2 users was 1214 (SD 374) minutes. In addition, the Fitbit Inspire 2 data recovery rate during the study period was 87.98% (2,837,881 minutes/3,225,600 minutes).

### Bedtime, TIB, Midpoint of Sleep, and Psychological State

**Figure 1:**
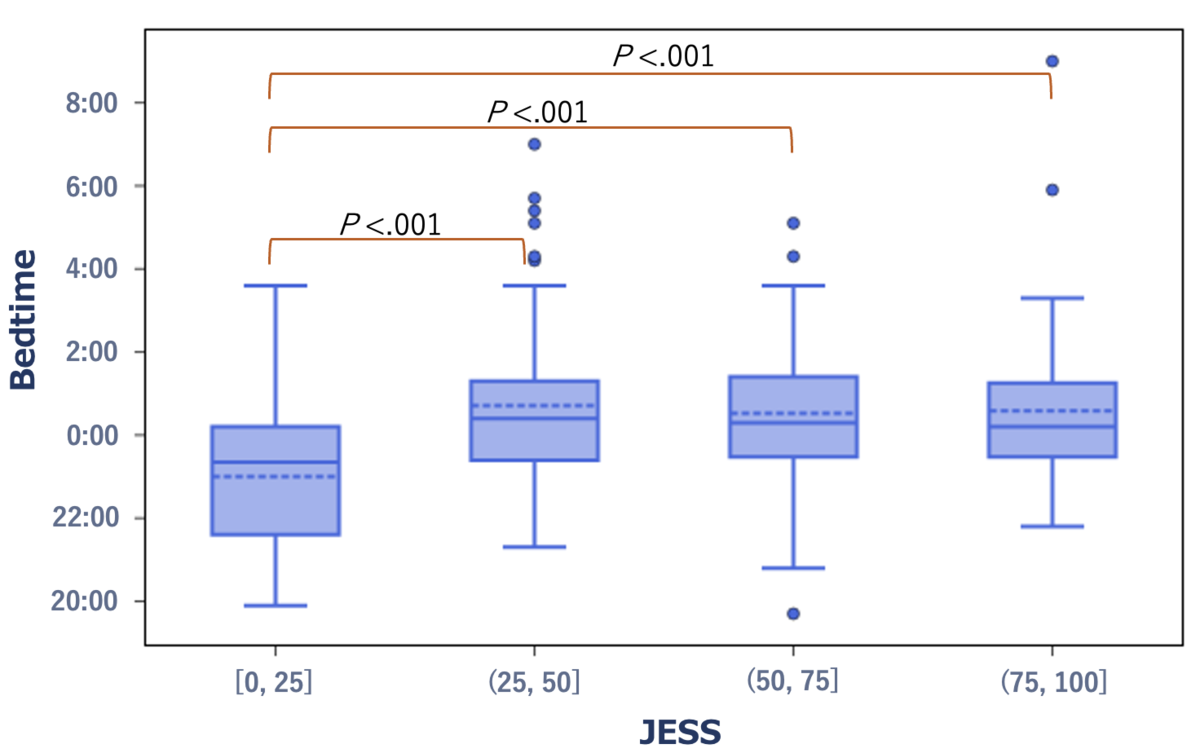
Box plot showing the distribution of bedtime in the low- and high-score groups of the JESS score. The dashed line in the box plot refers to the mean value and the whiskers refer to the minimum and maximum values within the IQR 1.5 range (same for subsequent figures). JESS: The Japanese version of the Epworth Sleepiness Scale.

**Figure 2:**
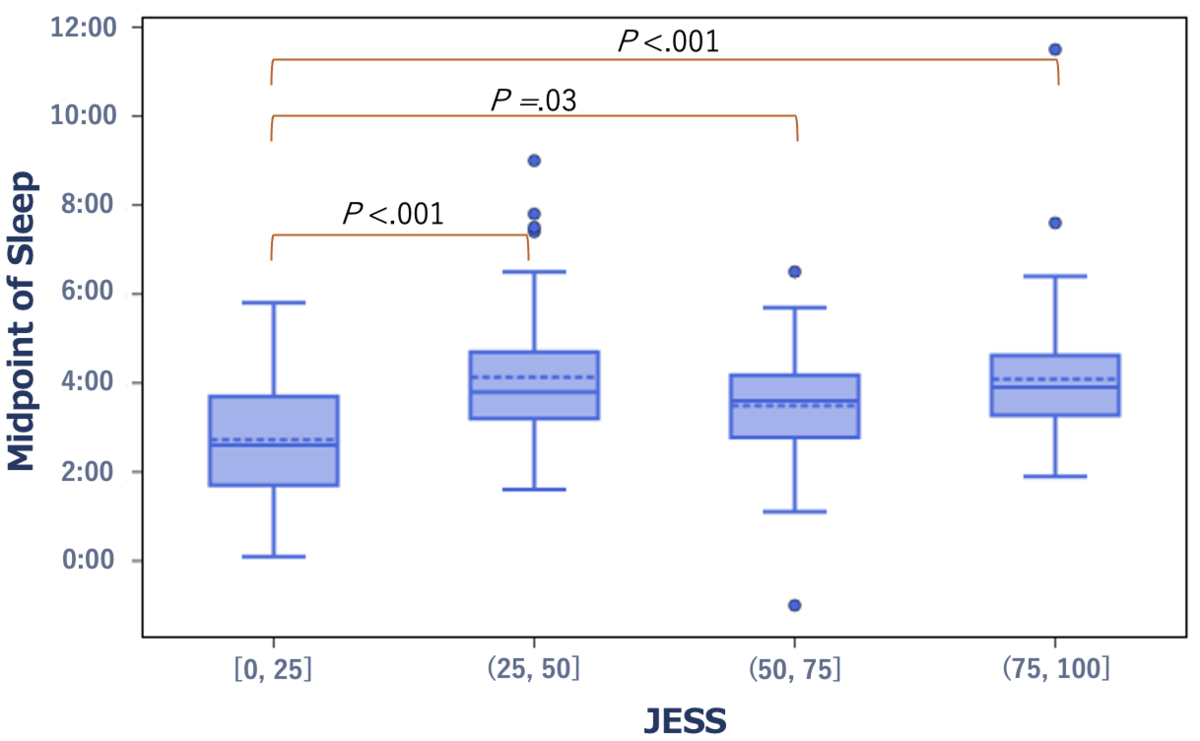
Box plot showing the distribution of the midpoint of sleep in the low- and high-score groups of the JESS score. JESS: The Japanese version of the Epworth Sleepiness Scale.

In terms of the relationship with JESS scores, compared with the group with high daytime sleepiness (third quartile to maximum; high-score group), the group with low daytime sleepiness (minimum to first quartile; low-score group) exhibited significantly earlier bedtime and midpoint of sleep (*r*=0.24, *P*<.001 and *r*=0.25, *P*<.001, respectively) (Figures 1 and 2). In the low JESS group, the median bedtime and midpoint of sleep were 11:21 PM and 2:36 AM, respectively. By contrast, in the high JESS group, the median bedtime and midpoint of sleep were 12:12 AM and 3:54 AM, respectively.

**Figure 3:**
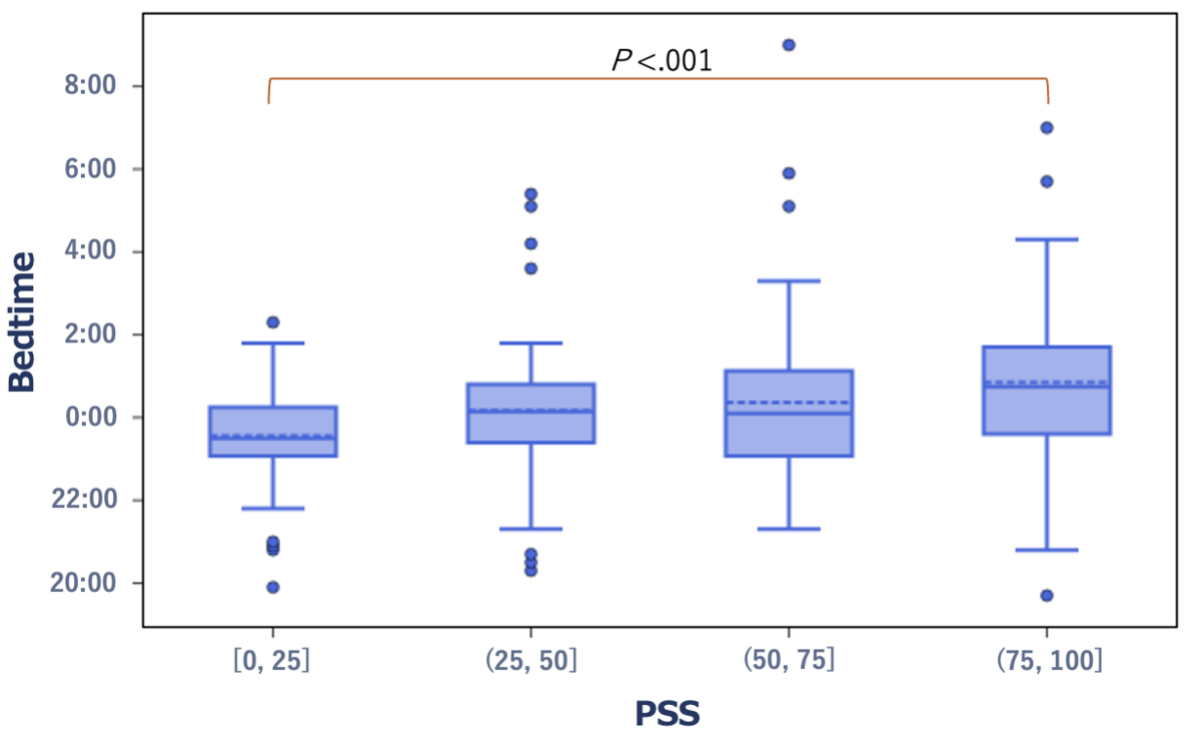
Box plot showing the distribution of bedtime in the low and high score groups of the PSS score. PSS: Perceived Stress Scale.

Regarding subjective stress, the median bedtime was 11:30 PM in the low PSS group and 12:45 AM in the high PSS group. Notably, the median bedtime was significantly earlier in the low PSS group (*r*=0.226, *P*<.001; Figure 3).

**Figure 4:**
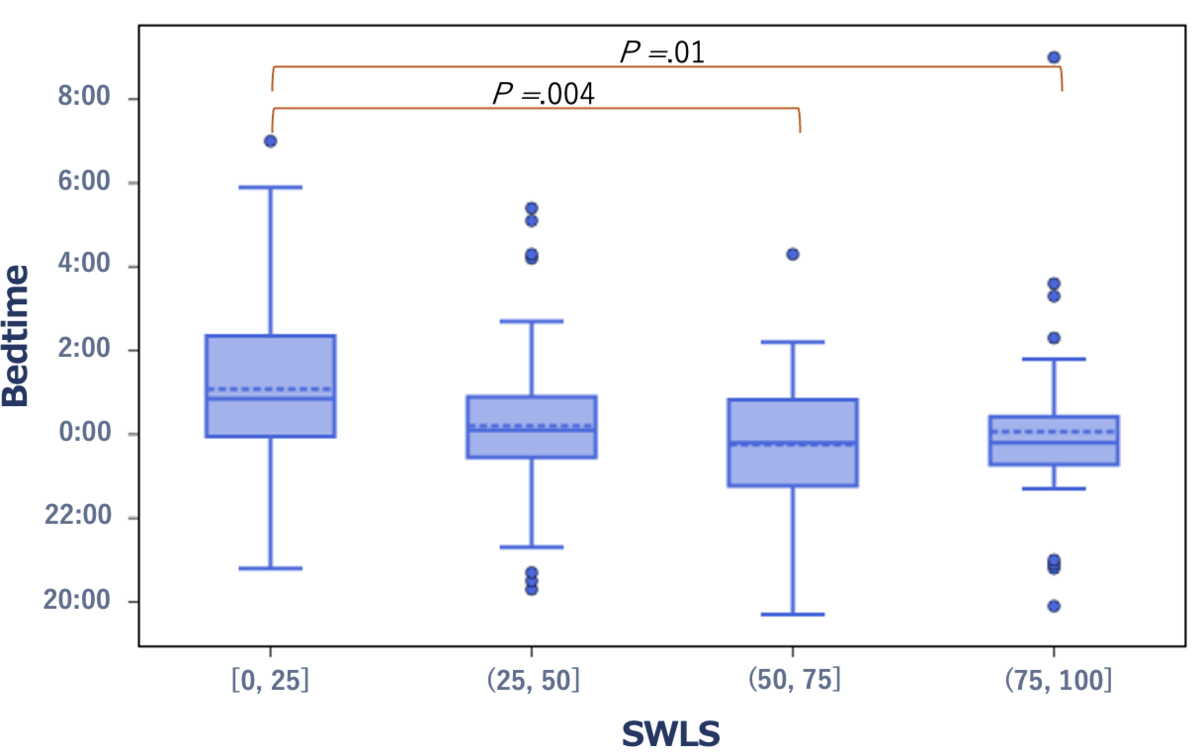
Box plot showing the distribution of bedtime in the low- and high-score groups of the SWLS. SWLS: Satisfaction With Life Scale.

**Figure 5:**
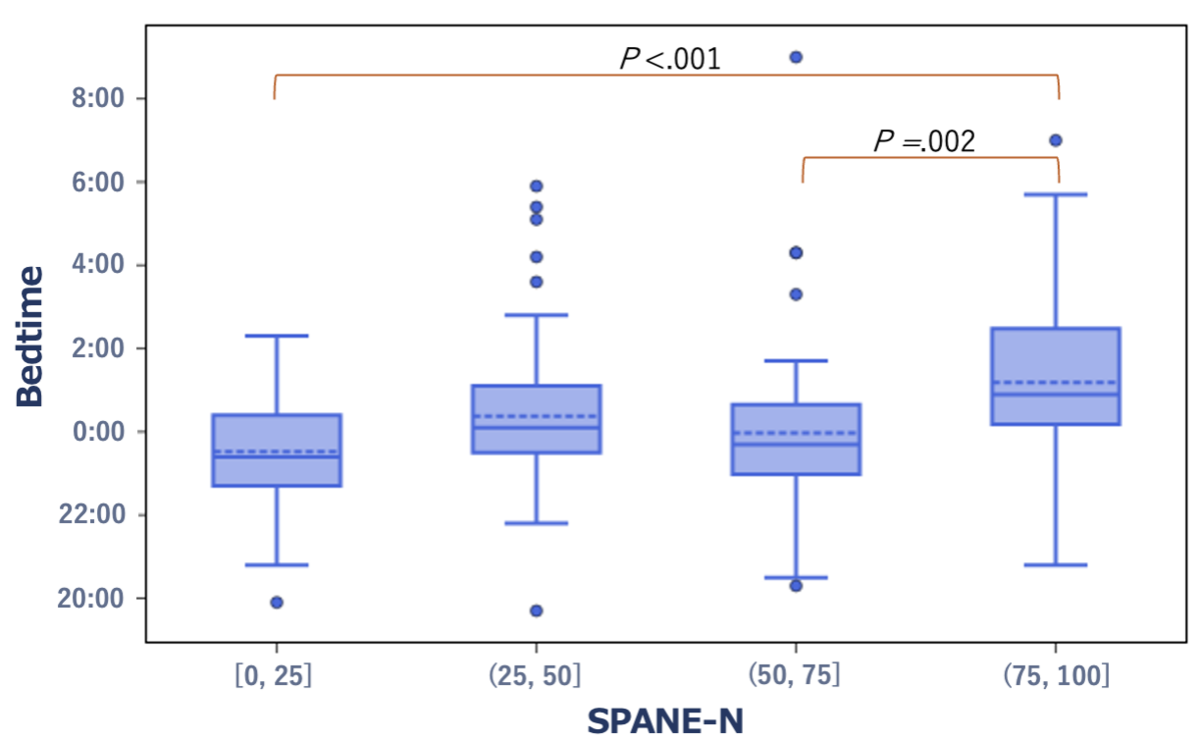
Box plot showing the distribution of bedtime in the low- and high-score groups of the SPANE-N. SPANE-J: The Japanese version of the Scale of Positive and Negative Experience; SPANE-N: SPANE-J negative experience.

In terms of well-being scores, the group with high SWLS scores and low SPANE-J negative experience (SPANE-N) scores exhibited significantly earlier bedtime (*r*=–0.23, *P*=.01 and *r*=0.23, *P*<.001, respectively; Figures 4 and 5). Specifically, the median bedtime was 11:48 PM and 11:24 PM in the high SWLS group and the low SPANE-N group, respectively. Conversely, in the low SWLS group and the high SPANE-N group, the median bedtime was 12:51 AM and 12:54 AM, respectively.

### Variability in the Midpoint of Sleep and Psychological State

**Figure 6:**
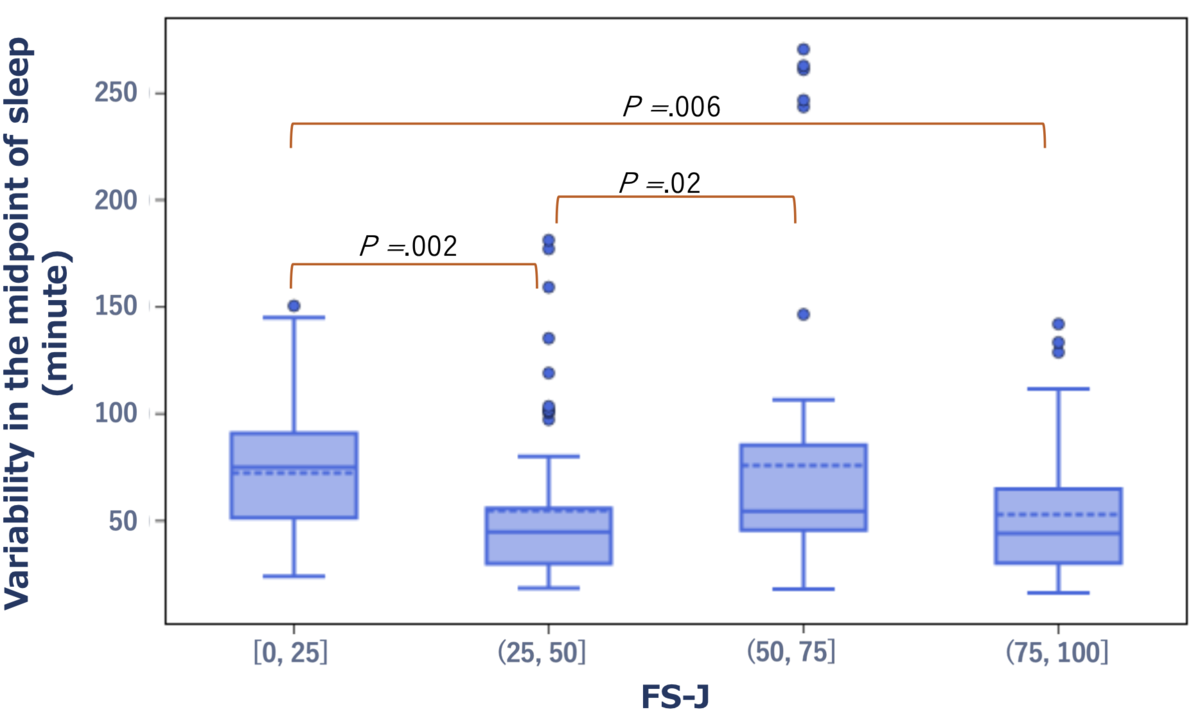
Box plot showing the distribution of variability in the midpoint of sleep in the low and high score groups of the FS-J. FS-J: The Japanese version of the Flourishing Scale.

**Figure 7:**
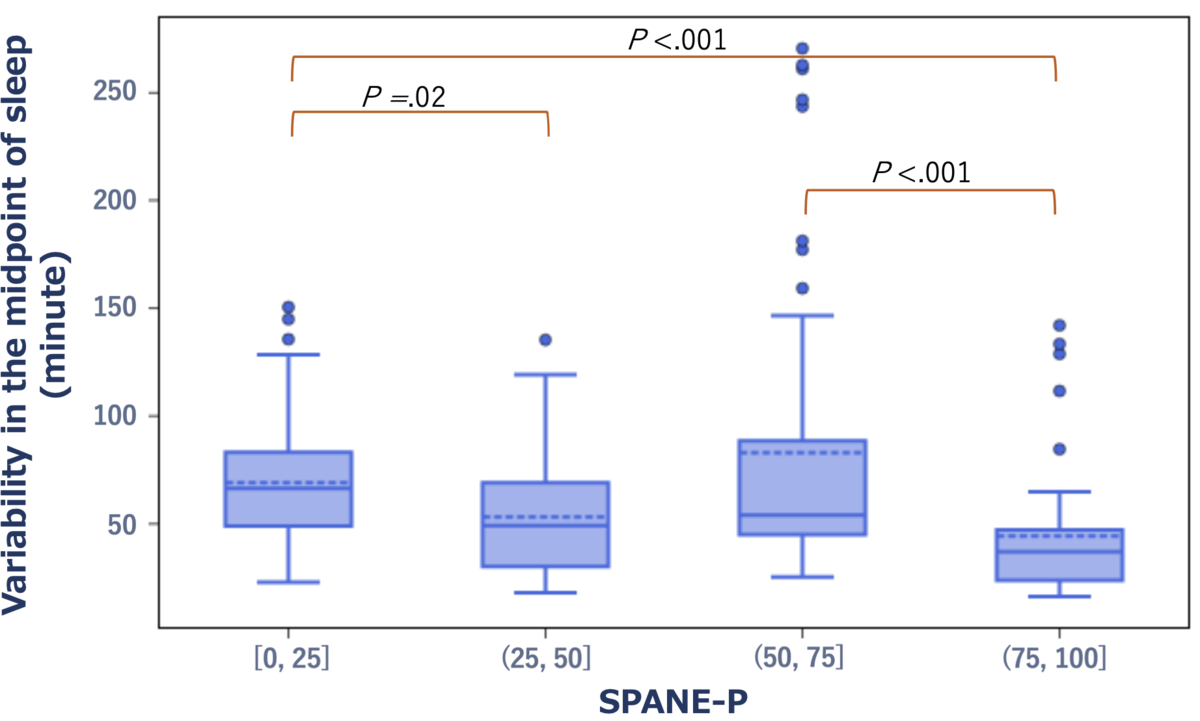
Box plot showing the distribution of variability in the midpoint of sleep in the low- and high-score groups of the SPANE-P. SPANE-J: The Japanese version of the Scale of Positive and Negative Experience; SPANE-P: SPANE-J positive experience.

The SD of the midpoint of sleep per week was significantly larger (|Hedge *g*|>0.5) in the group with low scores on the well-being scales, namely, the FS-J and the SPANE-J positive experience (SPANE-P), compared with the group with high scores (*r*=–0.11, *P*=.006 and *r*=–0.3, *P*<.001, respectively). Concerning the FS-J, the average SD of the midpoint of sleep per week was 75 minutes in the low-score group compared with 44 minutes in the high-score group. Similarly, based on the SPANE-P, the average SD was 67 minutes in the low-scoring group versus 37 minutes in the high-scoring group (Figures 6 and 7).

**Figure 8:**
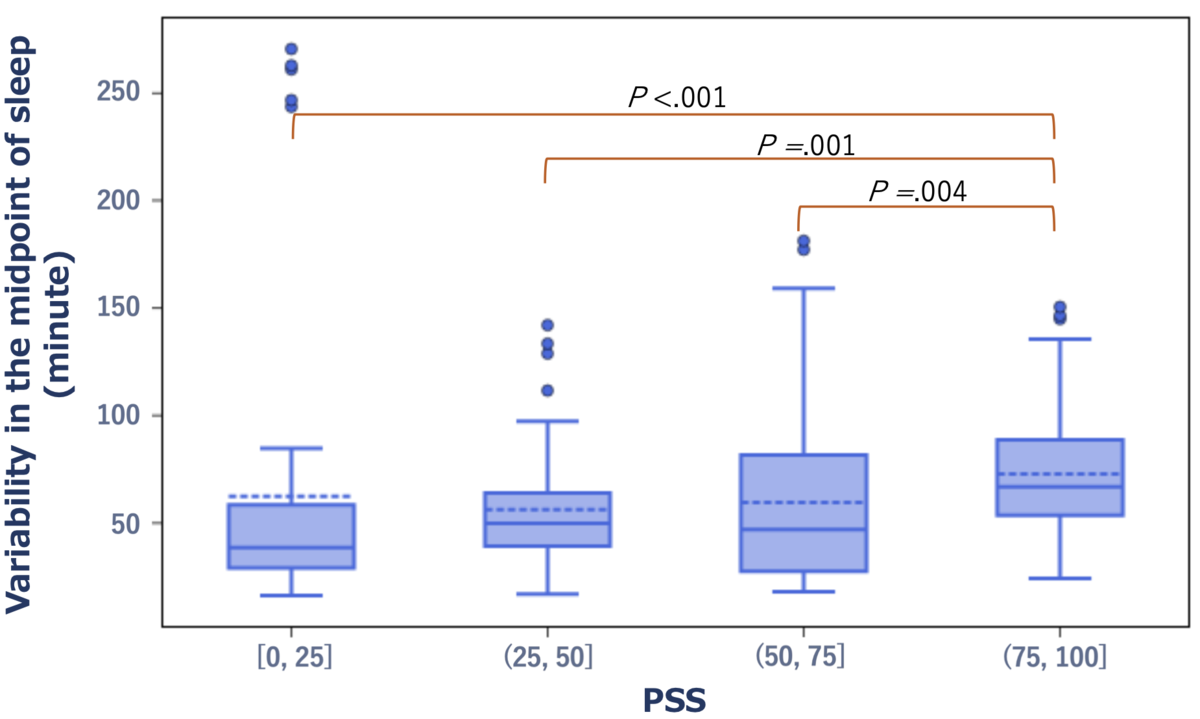
Box plot showing the distribution of variability in the midpoint of sleep in the low- and high-score groups of the PSS. PSS: Perceived Stress Scale.

Similarly, the SD of the midpoint of sleep was significantly greater in the group with high subjective stress as measured by the PSS compared with the group with low subjective stress (*r*=0.3, *P*<.001). Specifically, on the PSS, the median SD of the midpoint of sleep was 33 minutes in the low-score group versus 66 minutes in the high-score group (Figure 8).

### Sleep Duration, Sleep Efficiency, and Psychological State

There was no significant relationship between sleep duration and psychological state. However, the sleep efficiency was higher in the group with high scores on the PSS compared with the group with low scores (*r*=0.25, *P*=.006).

### HRV

HRV was assessed separately during daytime and bedtime. HRV during sleep, specifically the SDNN, was significantly higher in the high SWLS group compared with the low SWLS group (*P*=.003).

### EMA Response and Data Obtained From Fitbit Inspire 2

The intraclass correlation coefficients for the EMAs performed in this study were as follows: energy, 0.407; mood, 0.4075; and sleepiness, 0.3286.

The results of the multilevel analysis for the EMA response and data obtained from the Fitbit Inspire 2 are presented in Tables 3-5. Regarding intraindividual variability, energy levels tended to be higher when the deviation of heart rate during sleep on the previous day was lower (β=–.12, *P*<.001; Table 3). Furthermore, sleepiness levels tended to be lower on days with a higher deviation of time in bed (TIB; β=–.10, *P*<.001; Table 5).

Based on the interindividual evaluation of the relationships, participants with a lower deviation of the midpoint of sleep tended to have relatively higher energy (β=–0.26, *P*=.04; Table 3).

**Table 3:** Multilevel analysis of energy.^a^

Predictors	Energy		
	Estimates	CI	*P* value
Intercept	–0.12	–0.40 to 0.17	.42
Age	–0.09	–0.33 to 0.15	.48
Gender	0.22	–0.18 to 0.62	.29
Metabolic equivalents	0.02	–0.04 to 0.07	.54
Response time	0.03	–0.04 to 0.09	.40
Deviation of heart rate during sleep (intraindividual)	–0.12	–0.19 to –0.05	<.001
Midpoint of sleep (interindividual)	–0.26	–0.50 to –0.02	.04

^a^In addition to a random intercept, random effects were added to the coefficient of response time, metabolic equivalents, and deviation of heart rate during sleep for each user.

**Table 4:** Multilevel analysis of mood.^a^

Predictors	Mood		
	Estimates	CI	*P* value
Intercept	–0.08	–0.36 to 0.21	.60
Age	–0.02	–0.20 to 0.23	.87
Gender	0.13	–0.28 to 0.53	.54
Metabolic equivalents	0.04	–0.02 to 0.09	.19
Response time	0.08	0.03 to 0.13	.002
Deviation of the midpoint of sleep (intraindividual)	–0.04	–0.13 to –0.05	.38
Bedtime (interindividual)	–0.19	–0.41 to 0.03	.09

^a^In addition to a random intercept, random effects were added to the coefficient of response time, metabolic equivalents, and deviation of the midpoint of sleep for each user.

**Table 5:** Multilevel analysis of sleepiness.^a^

Predictors	Sleepiness		
	Estimates	CI	*P* value
Intercept	–0.16	–0.09 to 0.42	.21
Age	–0.09	–0.27 to 0.09	.33
Gender	–0.38	–0.74 to 0.02	.04
Metabolic equivalents	–0.09	–0.15 to 0.03	.005
Response time	0.07	–0.14 to 0.00	.04
Deviation of time in bed (intraindividual)	–0.10	–0.15 to –004	<.001

^a^In addition to a random intercept, random effects were added to the coefficient of response time, metabolic equivalents, and deviation of time in bed for each user.

## Discussion

### Principal Findings

In this study, we investigated the relationships between sleep indices and HRV, measured through the use of wearable devices, and the daily sleepiness, energy, and mood status assessed by EMA, along with stress and well-being questionnaires, in a cohort of 40 participants. The response rate of the participants was relatively high, and several significant associations were observed: greater variability of the midpoint of sleep was linked to lower well-being and higher stress levels, later bedtimes were associated with increased sleepiness and higher stress levels, and greater HRV during sleep was correlated with higher well-being.

The intraclass correlation coefficients of the EMA performed in this study exceeded 0.1 (energy, 0.407l; mood, 0.4075; and sleepiness, 0.3286). Hence, it can be concluded that the EMA VAS scores in this study captured not only within-individual (within-participant) variation but also psychological differences between individuals. The findings regarding the relationship between psychological state and sleep were consistent with previous studies, suggesting that wearable devices and EMA are valuable methods for quantifying both sleep and psychological state.

The results regarding mood and sleepiness were impacted by the level of activity around the response time, highlighting the importance of considering real-time information, including waking time, collected by both EMA and wearable devices when investigating the relationship between psychological state and sleep.

### Comparison With Prior Work

Regarding participation rates in studies using EMA, a meta-analysis on EMA compliance reported a typical response rate of 78%. Specifically, the average response rate was 92% for prompts 2-3 times per day for nonclinical participants, and 74% for prompts 3-4 times per day [42]. Therefore, we consider the EMA response rate in this study (1118/1280, 87.34%) to be within a reasonable range. Although not extensively detailed in this paper, it is noteworthy that this study also involved participants wearing the FreeStyle Libre in parallel. Therefore, it was significant that responses were collected without dropouts, despite participants likely having additional tasks compared with a typical study.

The results of this study revealed significantly higher HRV during sleep in individuals with higher scores on the SWLS. It is established that HRV is greater during high-quality sleep and that sleep-related HRV is compromised in individuals with sleep disorders and mental health conditions [43,44]. The findings of this study align with prior research indicating a relationship between sleep quality and well-being [3-5]. Moreover, the results demonstrated that individuals with earlier bedtimes exhibited lower levels of sleepiness, as measured by the JESS, and lower stress levels, as measured by the PSS. Previous studies have suggested that late bedtime is associated with daytime sleepiness and that sleep deprivation can impact daytime stress [27,45], which is consistent with our findings.

Furthermore, our study revealed that the SD of the midpoint of sleep was correlated with stress and well-being. In recent years, the issue of social jet lag—referring to the misalignment between an individual’s internal circadian rhythm and external social demands, often due to work and other societal factors—has gained recognition, particularly among urban office workers. This phenomenon has been noted in Japan as well [46,47]. Social jetlag has been increasingly associated with stress and well-being [48,49]. Our study findings among urban office workers similarly demonstrated a link between disruptions in sleep rhythm and stress/well-being, consistent with previous research on social jetlag. To our knowledge, there have been no studies investigating social jetlag in Japanese individuals using wearable devices or similar tools for objective assessment of sleep. This presents a promising area for future research, offering the opportunity to explore the relationship between social jetlag, sleep patterns, and various health outcomes in this population.

In this study, sleep efficiency was higher among participants reporting higher subjective stress. Typically, it is understood that when subjective stress is high, sleep efficiency and quality are lower [50,51]. However, the reasons for these results in our study remain unclear and warrant further investigation. It is plausible that the group reporting higher subjective stress may have experienced fatigue from work, leading them to fall asleep earlier. However, this is a hypothesis that requires further investigation in future studies.

Regarding the results of the EMA, later bedtimes were correlated with lower energy levels and mood. Previous research has indicated that poor sleep quality can impact positive emotions throughout the day [52], and the findings of our EMA analysis corroborate this trend [23]. Thus, the results of this study are consistent with prior findings in the literature.

### Limitations

This study had several limitations. First, as this was an exploratory study, formal power calculations were not conducted, and a practical sample size was adopted. A larger sample size might have enabled the detection of additional findings. Second, the study recruited a small number of participants from a single company in Japan. Given the presumably similar working environment of the participants, the generalizability of the results may be limited. For instance, sleep habits are likely to vary by occupation, as job stress and working hours are known to influence habitual sleep duration and quality [53]. To enhance the generalizability of the study results, it would be necessary to recruit participants from a diverse range of occupations with various lifestyles. Further, the study relied on the Fitbit Inspire 2 for the measurement using wearable devices, which may have limitations compared with more advanced monitoring technologies. Despite providing thorough instructions to participants before the study and conducting regular checks for defective products throughout the study period, intrinsic limitations such as device performance were unavoidable. Moreover, in this study, HRV was calculated from heart rate measurements with a sampling time of 5 seconds, which may be less accurate compared with using methods such as electrocardiography. Finally, the EMA in this study entailed participants answering questions 2 times a week, 4 times a day, to avoid placing excessive burdens on them. However, for instance, if evaluating social jetlag, it would be important to compare weekends and weekdays. Including additional days of the week could potentially yield more meaningful results. It is worth noting that Japanese individuals often tend to select answers that fall in the midpoint of the range in surveys [54]. This conservative response tendency might have limited their ability to reflect subtle differences in their condition from one day to the next in their EMA responses. Given the limited number of studies using EMA in healthy Japanese adults, it would be beneficial in future research to investigate the potential presence of a response tendency in this population. Furthermore, our EMA design had its limitations. Recognizing that all participants in this study were full-time workers who might often have difficulty responding immediately, we set the EMA notification time as a fixed time but provided a range of response deadlines. Indeed, a randomly scheduled EMA is preferable for evaluating highly variable psychological states. However, the accuracy of the EMA in this study is limited because of the use of scheduled EMAs, which can lead to participant learning and anticipation. In our study, analysis was conducted based on response time rather than the sending time of the EMA. This approach ensured that regardless of any significant discrepancy between the sending and response times, the answers were elicited at the time of the response. However, it is important to note that this approach can introduce bias by only collecting data when participants choose to respond. Nevertheless, considering that the median difference between the sending and response times was 11.8 minutes, we believe that this issue was not significant in our study.

### Conclusions

In this study, the combination of objective assessment of sleep using a wrist-worn device worn by participants, along with subjective symptom assessment through EMA, provided valuable insights into the relationship between sleep and stress/well-being, particularly considering daily variations in sleep patterns. For example, increased sleep variability, characterized by unstable bedtime or midpoint of sleep, was linked to elevated stress levels and diminished well-being. Furthermore, improved sleep metrics, such as lower heart rate during sleep and earlier average bedtime, correlated with enhanced daytime energy levels. Conducting broader-scale studies, such as those on social jetlag, using a methodology akin to that of this study and encompassing a wider array of occupations, could provide valuable insights.

## Appendix

Multimedia Appendix 1 Histograms depicting the groups formed within each quartile by the population at each score.

## Abbreviations

BDHQ: Brief-Type Self-Administered Diet History Questionnaire
EBQ: Eating Behavior Questionnaire
EMA: ecological momentary assessment
FS-J: The Japanese version of the Flourishing Scale
HRV: heart rate variability
JESS: The Japanese version of the Epworth Sleepiness Scale
METs: metabolic equivalents
PSS: Perceived Stress Scale
SDNN: SD of the NN intervals
SPANE-J: The Japanese version of the Scale of Positive and Negative Experience
SPANE-N: SPANE-J negative experience
SPANE-P: SPANE-J positive experience
SWLS: Satisfaction With Life Scale
TIB: time in bed
VAS: Visual Analog Scale

## Data Availability

All data generated and analyzed are included in this article and a previously published article [38,39]. Raw data sets analyzed during this study are available from the corresponding author on reasonable request.
