# Supplementary material for: Assessment of Stress and Well-Being of Japanese Employees Using Wearable Devices for Sleep Monitoring Combined With Ecological Momentary Assessment: Pilot Observational Study
Source: JMIR Form Res. 2024 May 2;8:e49396. doi: 10.2196/49396 (PMC11099815; doi:10.2196/49396)

Figure S1 Histogram of what groups were formed in each quartile by the population of scores on the Japanese version of the Flourishing Scale (FS-J).

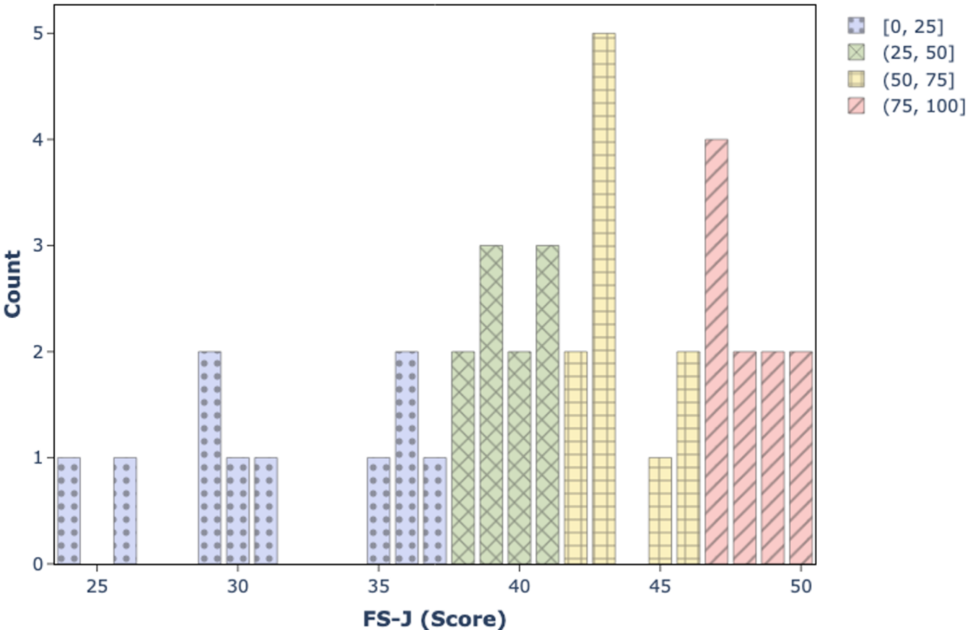

Figure S2 Histogram of what groups were formed in each quartile by the population of scores on the Japanese version of the Epworth Sleepiness Scale (JESS).

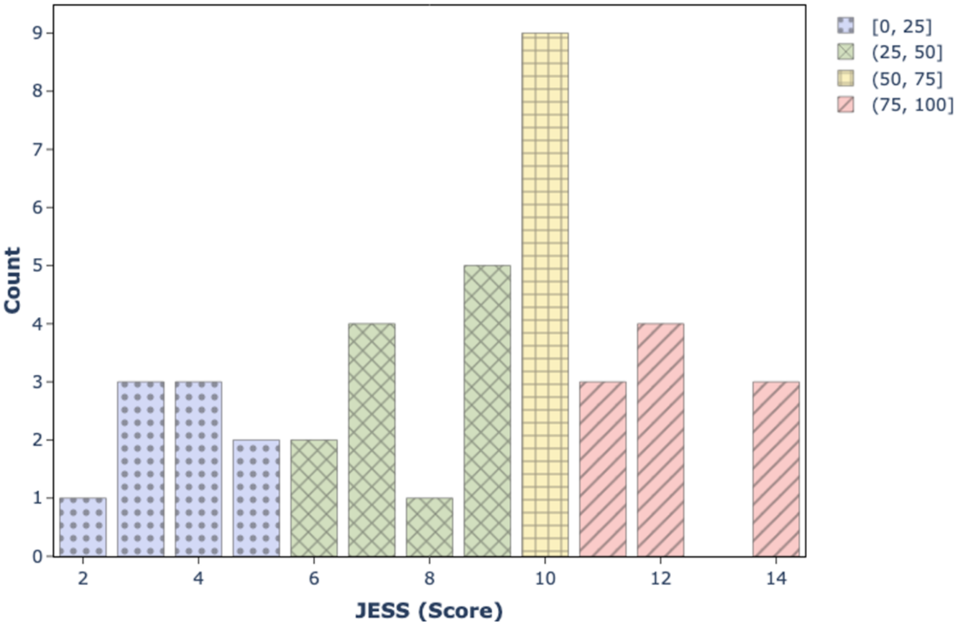

Figure S3 Histogram of what groups were formed in each quartile by the population of scores on the Perceived Stress Scale (PSS).

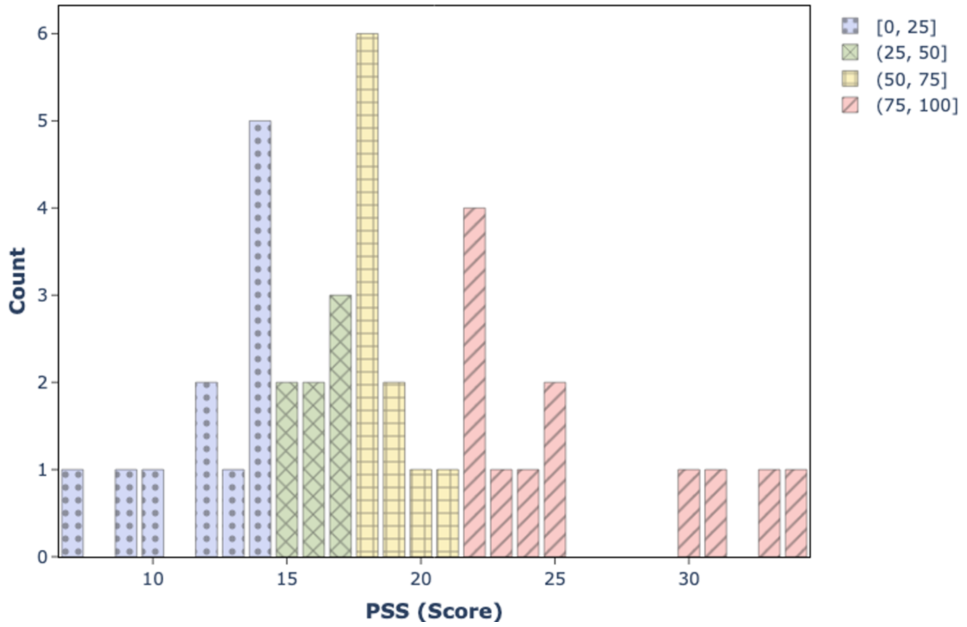

Figure S4 Histogram of what groups were formed in each quartile by the population of scores on the SPANE-J positive experience (SPANE-P).

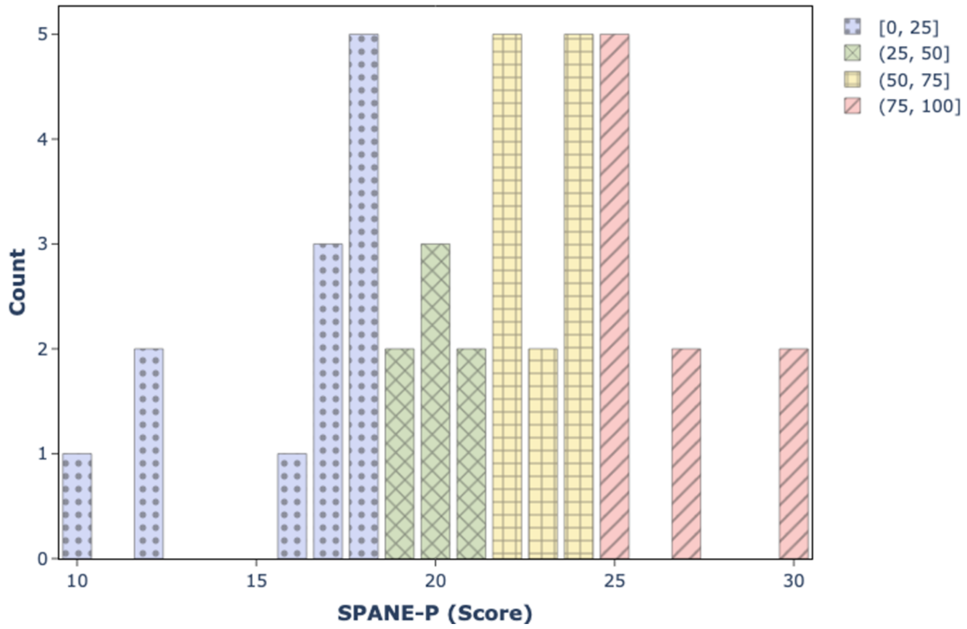

Figure S5 Histogram of what groups were formed in each quartile by the population of scores on the SPANE-J negative experience (SPANE-N).

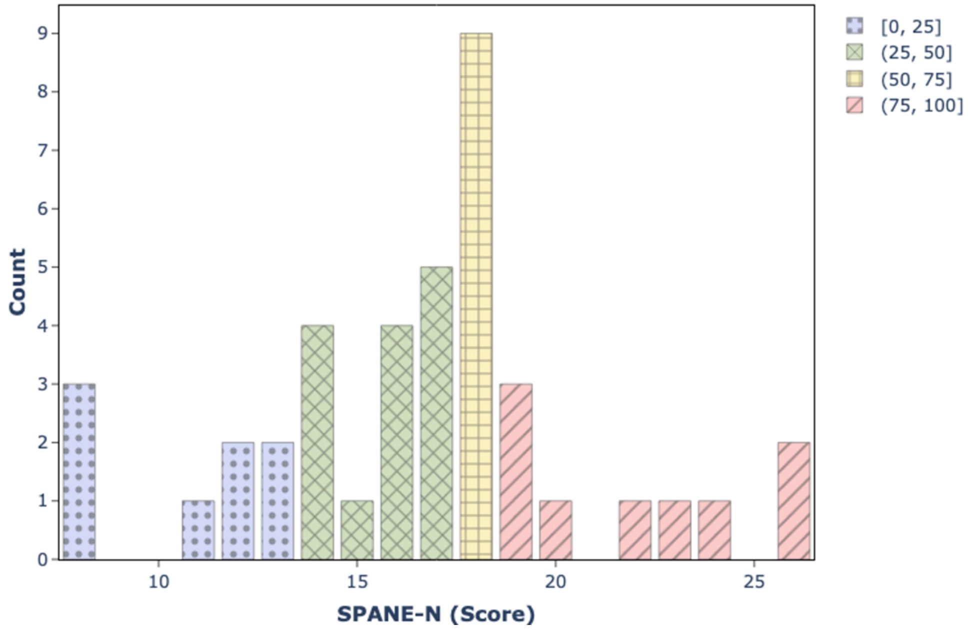

Figure S6 Histogram of what groups were formed in each quartile by the population of scores on the Satisfaction-With-Life Scale (SWLS).

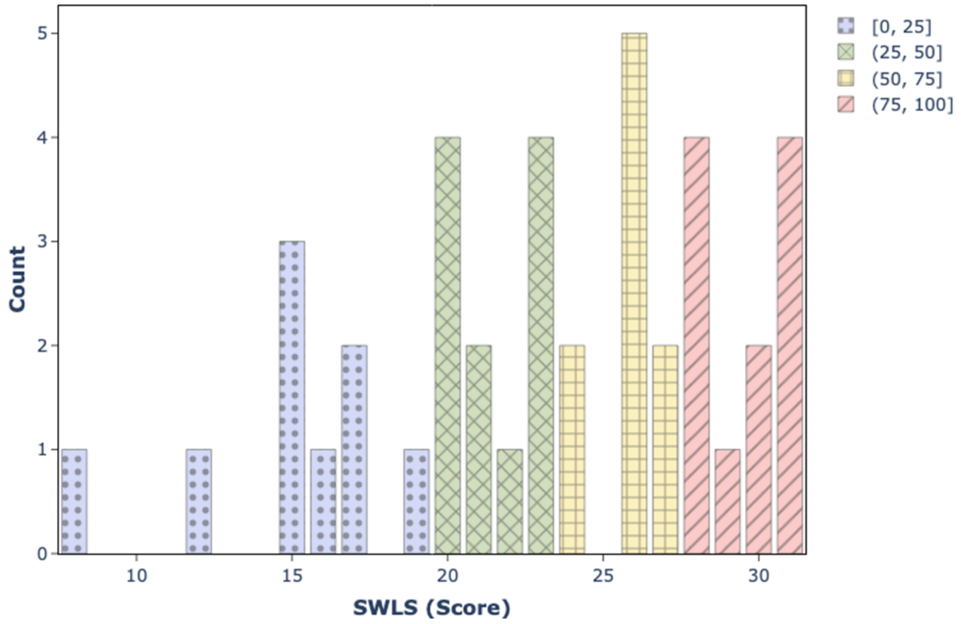

Supplement: Multimedia Appendix 1 [file formative_v8i1e49396_app1.pdf]
